# Effect of social participation on the trajectories of activities of daily living disability among community-dwelling older adults: a 7-year community-based cohort

**DOI:** 10.1007/s40520-024-02758-y

**Published:** 2024-05-07

**Authors:** Cai Wen, Shuangyuan Sun, Limei Huang, Yanfei Guo, Yan Shi, Shige Qi, Guomei Ding, Zhiqin Wen, Jiaqi Wang, Ye Ruan, Qi Zhao

**Affiliations:** 1 School of Public Health, NHC Key Laboratory of Health Technology Assessment, Fudan University, Shanghai, China; 2 Shanghai Municipal Center for Disease Control and Prevention, Shanghai, China; 3 Songjiang Center of Disease Prevention and Control, Shanghai, China; 4 National Center for Chronic and Noncommunicable Disease Control and Prevention, Chinese Center for Disease Control and Prevention, Beijing, China; 5 Zhongshan Community Health Care Center, Songjiang District, Shanghai, China; 6 Yexie Community Health Service Center, Songjiang District, Shanghai, China

**Keywords:** BADL/IADL, Social participation, Group-based trajectory model, Older adults, China

## Abstract

**Introduction:**

Studies examining the effects of social participation on activities of daily living (ADL) disability are still scarce.

**Aim:**

To assess the reciprocal relationship between ADL disability trajectories and social participation among older Chinese people aged ≥ 60 years.

**Methods:**

This study included 2976 participants aged ≥ 60 years in six waves of a community-based survey from 2015 to 2022. Basic activities of daily living (BADL) and instrumental activities of daily living (IADL) were used to assess the ADL disability in each survey. Social participation was assessed by involvement in four social activities and an extensive social participation score. Group-based trajectory modeling was used to identify potential heterogeneity in longitudinal changes over 7 years and explore associations between baseline predictors of group membership and these trajectories.

**Results:**

Two BADL disability trajectories were identified: stable (94.8%) and increase (5.2%). Additionally, three IADL disability trajectories were distinguished: stable (73.2%), moderate (20.2%), and increase (6.6%). After controlling for the potential covariates, each point increase in the extensive social participation score correlated with a 17% decrease in the odds of older individuals belonging to the increase BADL trajectory group (OR = 0.83, 95% CI = 0.68–1.00). For IADL, it decreased the odds of being assigned to the moderate trajectory group by 16% (OR = 0.84, 95% CI = 0.75–0.95) and to the increase trajectory group by 23% (OR = 0.77, 95% CI = 0.64–0.93).

**Conclusions:**

Higher levels of social participation among older individuals were more likely to be classified as stable trajectories in both BADL and IADL. Increased participation in social activities by community-dwelling elderly adults may promote healthy aging.

**Supplementary Information:**

The online version contains supplementary material available at 10.1007/s40520-024-02758-y.

## Introduction

Population aging, associated with elevated risks of chronic diseases stemming from physical or cognitive disability and mortality, poses significant challenges to contemporary healthcare, the economy, and society at large, making it a global public health priority [1, 2]. The proportion of the world’s population aged 60 years and older will dramatically double to 2.1 billion in 2050, accounting for 22% of the global population [3], which indicates that there will be more disabled older adults in the future. Disability can be defined as difficulties performing socially defined roles and tasks commonly assessed by activities of daily living (ADL) [4]. ADL encompasses two categories: basic activities of daily living (BADL), which pertain to self-care activities like bathing, dressing, and using the toilet, and instrumental activities of daily living (IADL), which involve more complex tasks such as cooking, managing finances, and shopping [5, 6]. An estimation indicated that the number of older people with ADL disabilities in China is projected to increase from 17.9 million in 2015 to 96.2 million in 2060 [7]. The steady increase in ADL disabilities poses a significant challenge to affected individuals, families, and healthcare systems [8]. Previous studies have indicated that disability is a complex and dynamic process, with high recovery rates and frequent transitions between states [9, 10]. Good quality of life and vigorous physical activity significantly contribute to ADL function, and early interventions hold promise in mitigating the rate of decline [11–13]. Trajectories, as a result of ongoing interactions between individuals and their environment, not only intuitively demonstrate the trend of improvement in ADL but also identify distinct subgroups of individuals with similar patterns of ADL development rather than treating the population as homogeneous [14]. Therefore, it is critical to understand disability trajectories and associated factors in older adults for implementing early targeted interventions [15, 16].

For older people, social participation is regarded as an indicator of successful and healthy aging. Social participation, including leisure activities, meeting friends, and physical exercise, empirically promote older individuals’ well-being, quality of life, and favorable health outcomes [17–19]. The existing literature indicates a specific association between social participation and ADL disability. Results from longitudinal studies conducted in the United States [20], China [21], South Korea [12], and Japan [22] demonstrated that increased social participation helped maintain the basic and instrumental daily living abilities of older adults. In China, the social and structural context of social participation among older adults differs from that of developed countries. This difference is rooted in the cultural tradition of familism, which emphasizes strong bonds and mutual support within family and kinship networks [23]. Unlike the well-organized social activities prevalent in civil societies, social participation among Chinese older adults primarily revolves around self-organized activities, such as group dancing, card games, mahjong, and other cultural and leisure-time activities [24]. Meanwhile, due to significant heterogeneity in the dynamic development of daily life self-care abilities among the elderly population in China [8, 16], there is a lack of research utilizing trajectory models to investigate the association between social participation and the trajectory of ADL disability over time. The underlying mechanisms of this association remain unclear.

Therefore, we had two main aims in the current study involving longitudinal data from a 7-year prospective cohort study among community-dwelling older adults aged 60 and above in Shanghai from 2015 to 2022: (1) to investigate the dynamic BADL and IADL disability trajectories via group-based modeling (GBTM); (2) to explore the association of social activity participation levels and various types of social participation on these trajectory changes.

## Methods

### Study subjects

The participants were from the population-based prospective cohort in Songjiang District, Shanghai [25]. We utilized a multi-stage random sampling method that selected two of eleven streets. Following this, ten communities were randomly chosen from the two streets. Based on the annual census data of Shanghai in 2014, we identified 16,809 permanent residents aged 60 or older in these ten village communities [25]. Ultimately, we recruited 4050 permanent residents aged 60 or older for an inquiry in mid-2015. Subsequently, we conducted five follow-up assessments in 2017, 2018, 2019, 2021, and 2022. The number of participants at the baseline and the five follow-up waves was as follows: 4050 (2015), 3990 (2017), 3835 (2018), 3566 (2019), 3390 (2021), and 3184 (2022).

**Fig. 1:**
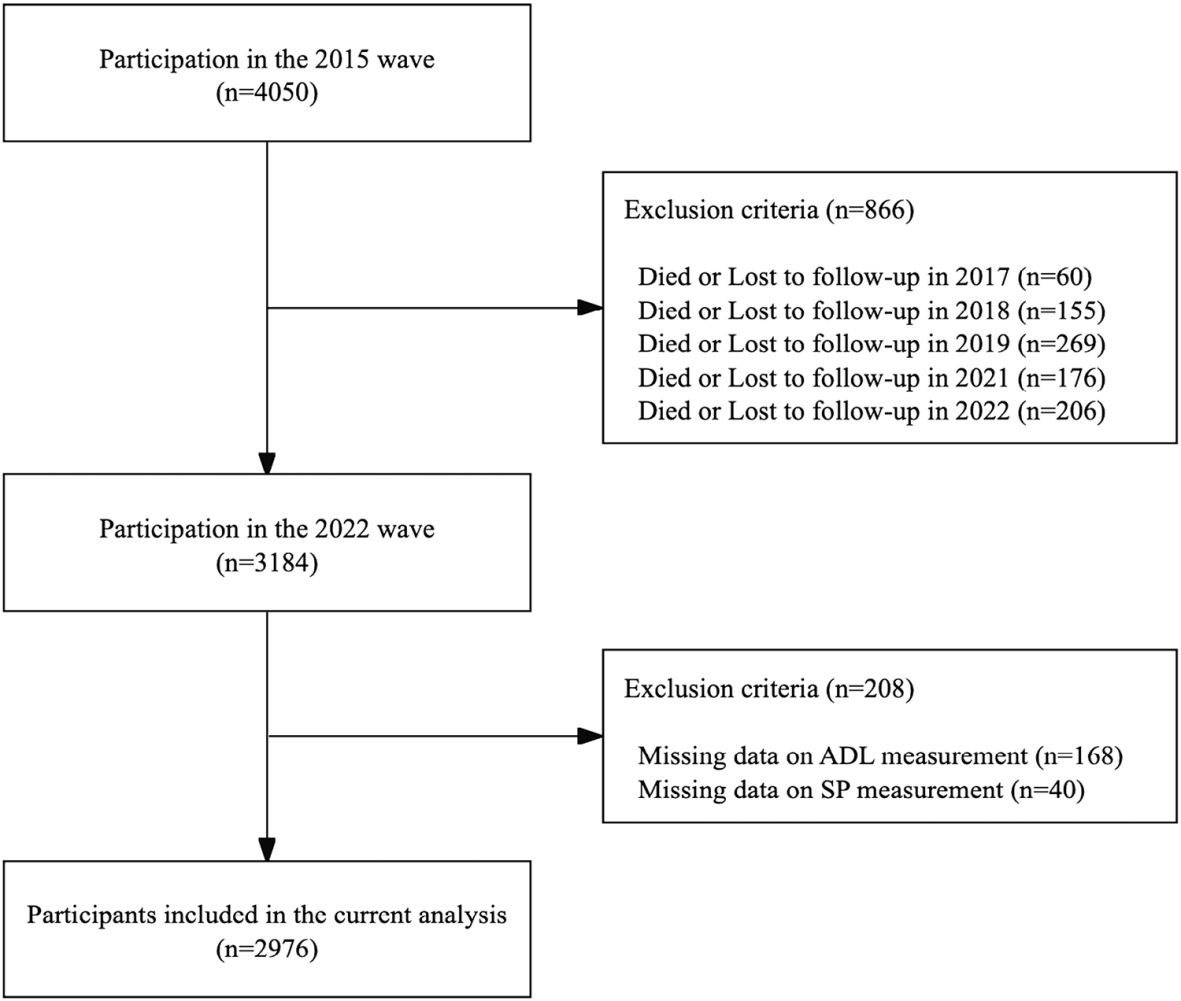
Flowchart of sampling

For this study, three inclusion criteria were used to restrict the survey data: (1) respondents who were ≥ 60 years old at baseline; (2) had completed all six-wave surveys; and (3) had no missing data on indicators of ADL and social participation. A total of 2976 respondents were involved in this study (Fig. 1). This study obtained ethical approval from the Ethics Review Committee of the Chinese Center for Disease Control and Prevention (Approval Number: 201620). Participants joined the study by completing the questionnaire and the written informed consent form.

### Ascertainment of activities of daily living disability

Disability was measured by the ADL scale [5, 6], which is composed of two parts: basic activities of daily living (BADL) and instrumental activities of daily living (IADL). BADL included the seven items (bathing, eating, dressing, continence, toileting, getting in and out of bed, and indoor transferring). IADL was measured by the eight items (preparing meals, shopping for groceries, managing money, making phone calls, doing light housework, doing heavy housework, getting to places outside walking distance, and managing medications).

The response to each question was graded into four categories: (1) no difficulty; (2) difficulty but without needing help; (3) needing some help; and (4) needing complete help. If the respondents selected the first two response categories for an activity, they were categorized as independently performing the activity and assigned a value of 0. Conversely, dependence was assigned a value of 1. The total BADL and IADL scores ranged from 0 to 7 and 0 to 8, respectively, with 0 representing no disability in BADL or IADL. The same questions were used in each wave of data collection.

### Assessment of social participation

According to previous studies, social participation is a broad concept and can involve many dimensions [26–28]. In the current study, given the social context for the social participation of Chinese older adults, social participation was derived based on four types of social activities at baseline, including engagement in formal social activities (such as group activities, senior associations, elderly educational, or religious activities organized by community organizations, neighborhood committees, or institutions), informal group leisure activities (cards/mahjong), neighborly interactions (lasting more than 10 min), and physical activities (involving fitness activities lasting more than 10 min, such as walking, sports, jogging, qi gong, or agricultural work). All chosen types of social participation were binary variables, with a score of 1 assigned if participation occurred within the past 12 months and 0 if it did not.

To comprehensively assess the social participation of older adults, we constructed a composite measure termed "extensive social participation" by summing the scores for the four subdimensions of social activities mentioned above, with a higher score representing a greater degree of social participation (ranging from 0 to 4). Furthermore, the extensive social participation scores were categorized into two levels (high and low) based on the median score [23, 26].

### Covariates

Age, sex, marital status, education, work status, living arrangements, and economic status were assessed by self-reporting at baseline. Smoking status was categorized as current smokers and never smokers. Drinking status was divided into drinking and never drinking. BMI was calculated as weight in kilograms divided by height in meters squared. According to WHO cutoff points [29], BMI was categorized as underweight (< 18.5 kg/m^2^), normal (18.5–24.9 kg/m^2^), overweight (25–29.9 kg/m^2^), and obese (≥ 30 kg/m^2^). Cognitive status was assessed using the AD8 screening questionnaire; participants with an AD8 score ≥ 2 were categorized as having probable cognitive impairment, whereas those with an AD8 score < 2 were classified as cognitively normal. Participants were asked if they were ever diagnosed with any of the 15 chronic conditions, namely, hypertension, diabetes, heart diseases, stroke, bronchitis (or emphysema), asthma, chronic kidney disease, tuberculosis, glaucoma, cataracts, arthritis, cervical spondylosis, herniated discs, deafness (or hearing loss), urinary incontinence. The number of chronic diseases was calculated by summing up the diagnosed diseases and then categorized into three groups: 0 (no chronic disease), 1 (one chronic disease), and 2 (at least two chronic diseases) based on tertiles.

### Statistical analysis

To determine the best-fitting discrete number and pattern of ADL score trajectories, we used group-based trajectory modeling (GBTM), which is increasingly applied in healthy aging research [30, 31]. The time metric was the years since baseline (2015–2022), and six waves of ADL scores were used to estimate the trajectory. Since the ADL score was a count variable with excess zero counts, we implemented the GBTM with a zero-inflated Poisson distribution using the CrimCV package [32]. The model fit was identified by the Bayesian information criterion (BIC) value and the average posterior probability of each group. The smaller the BIC value, the better the fit of the trajectory model; the threshold of the average posterior probability is 0.7, and values ≥ 0.7 indicate an acceptable model fit. Each participant was assigned to a trajectory based on posterior classification probabilities, ensuring that the proportion of individuals within each subgroup relative to the total population was greater than 5% [33].

Baseline characteristics of the study population are described as numbers and proportions (%) for categorical variables, whereas continuous variables were described as mean ± standard deviation (SD) or median interquartile range (IQR). The Mann–Whitney U test, Kruskal–Wallis H test, or Chi-Square test was employed for BADL/IADL disability trajectory group comparisons. We used logistic regression for binary variables and multinominal logistic regression for ordinal variables. Additionally, we examined the robustness of the estimation by fitting different models. Model 1 was a univariate model, and we adjusted sociodemographic characteristics in Model 2, including age, sex, marital status, educational attainment, work status, living arrangements, and bathroom facilities. Model 3 incorporated adjustments for all covariates and further considered smoking status, alcohol consumption, BMI, cognitive status, and the number of chronic diseases. We calculated the crude odd ratios (cORs) and adjusted odd ratios (aORs) with 95% CIs for the effect of social participation on ADL disability.

All analyses were performed using R software, version 4.2.2 (R Project for Statistical Computing), and a two-sided *P* < 0.05 was considered statistically significant for all analyses.

## Results

### BADL/IADL disability trajectory models

Among 1–4 group models, the 2-group model had the lowest BIC values and relatively high AvePP for each group in BADL, indicating its best fit. In IADL, although the difference in BIC values between the 3-group and 4-group models was marginal, the 4-group model contained a trajectory group with an AvePP of only 0.695, and the proportion of this group was only 4.5%, potentially suggesting insufficient representation [34], which did not meet the acceptable criteria of GBTM. In contrast, the performance of all indicators in the 3-group model met the requirements, indicating superior goodness of fit (Table 1).

**Table 1:** Estimates of group-based trajectory model for BADL/IADL disability trajectories

	Class 1	Class 2	Class 3	Class 4
BADL				
AIC	5175.46	4637.38	4645.09	4632.82
BIC	5222.20	4738.66	4800.89	4843.15
AvePP	1.000	0.980/0.983	0.964/0.909/0.791	0.972/0.845/0.676/0.743
Log-likelihood	−2581.73	−2305.69	−2302.55	−2289.41
IADL				
AIC	20,901.26	18,545.72	18,028.76	17,924.00
BIC	20,948.00	18,646.99	18,184.56	18,134.33
AvePP	1.000	0.959/0.975	0.907/0.836/0.949	0.695/0.894/0.880/0.931
Log-likelihood	−10,444.63	−9259.86	−8994.38	−8935.00

*BADL* basic activity of daily living, *IADL* instrumental activity of daily living, *AIC * Akaike information criteria, *BIC * Bayesian information criterion, *BIC*  Bayesian information criteria, *AvePP * average posterior probability

**Fig. 2:**
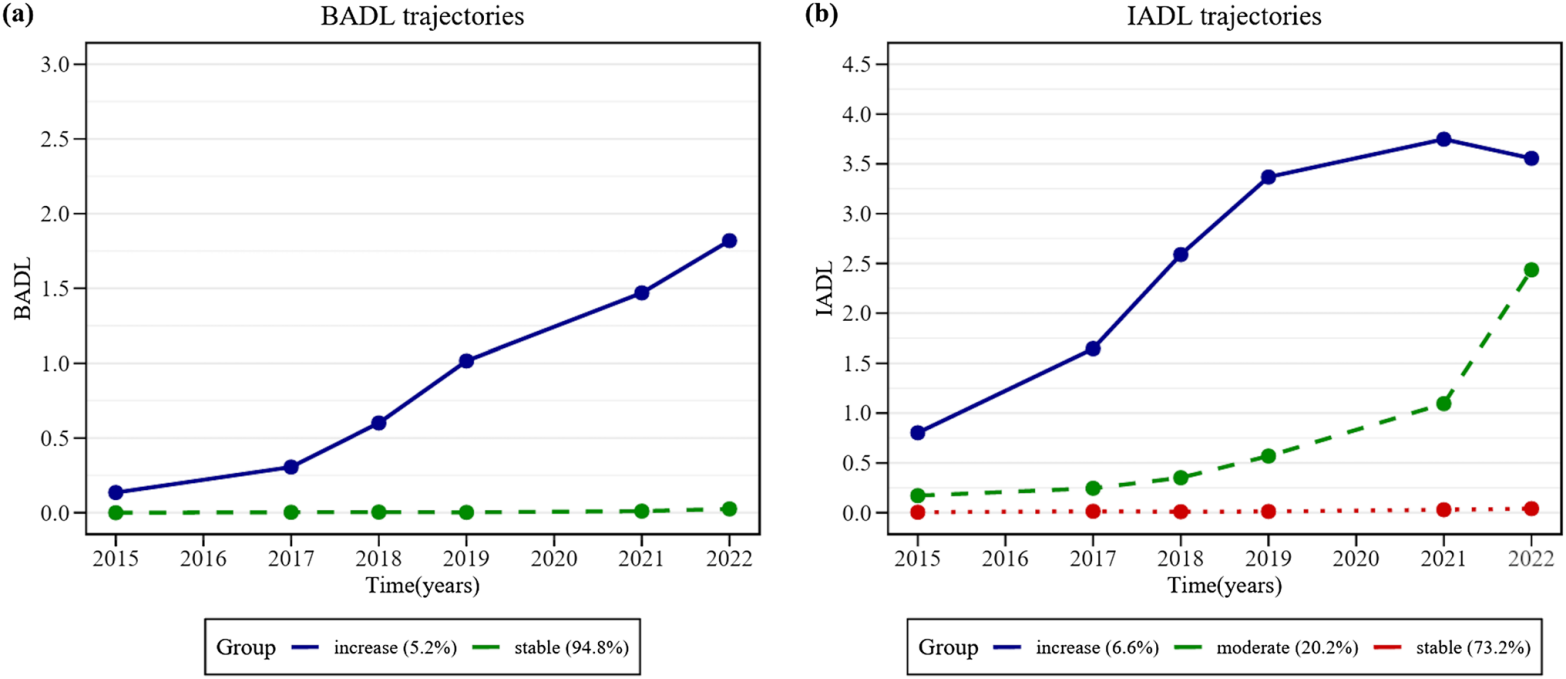
Disability trajectories of **a** BADL and **b** IADL for stable, moderate and increase groups BADL, basic activity of daily living; IADL, instrumental activity of daily living

Thus, there were two distinct groups in BADL identified over the 7-year follow-up (Fig. 2a): (1) a group that maintained a low BADL score throughout (stable group, 94.8%); (2) a group that commenced with relatively high BADL scores and then experienced a gradual yet accelerating increase in disability (increase group, 5.2%). In IADL, there were three distinct groups identified over the 7-year follow-up (Fig. 2b): (1) a group that maintained a low IADL score throughout (stable group, 73.2%); (2) a group that started with moderate IADL scores, experienced a sustained but gradual increase in disability over the first four waves, and then a sharp escalation (moderate group, 20.2%); (3) a group that initiated with the highest IADL scores, underwent a continuous substantial increase in disability over the first four waves, and then exhibited a gradual plateauing (increase group, 6.6%).

### Baseline characteristics

Table 2 presents the baseline characteristics of participants. A total of 2976 eligible older adults were included, with an average age of 68.1 years (standard deviation 6.2 years). Among them, 57.8% were female, and 83.1% were married. In terms of educational attainment, 56.5% had no formal education. Nearly half (46.4%) were retired. The prevalence of current smoking and drinking was 21.4% and 19.3%, respectively. Over half (58.0%) had a normal body mass index (BMI). Very few (4.8%) had cognitive impairment. Regarding chronic diseases, 31.4% had none, 36.4% had one, and 32.2% had two or more. For different forms of social participation, 35.6% engaged in organized activities, 24.6% played cards/mahjong, 91.0% interacted with neighbors, and 80.9% participated in physical activities. Overall, the median extensive social participation score was 2.00 with an interquartile range of 1.00. At least one ADL difficulty was present in 6.1% of participants (Supplementary Table 1). The characteristics of the participants stratified by social participation were shown in Supplementary Tables 2 and 3.

**Table 2:** Participant characteristics at baseline by BADL/IADL disability trajectory groups ^a^

Variable	Total (n = 2976)	BADL		*P-trend* ^*^	χ^2^/W	IADL			P-trend^*^	χ^2^/H
		Stable (n = 2822)	Increase (n = 154)			Stable (n = 2179)	Moderate (n = 600)	Increase (n = 197)		
Extensive social participation (median (IQR))	2.00 (2.00, 3.00)	2.00 (2.00, 3.00)	2.00 (1.00, 3.00)	0.001	26.714	2.00 (2.00, 3.00)	2.00 (2.00, 3.00)	2.00 (1.00, 3.00)	< 0.001	51.247
Extensive social participation level (%)										
Low	486 (16.3)	438 (15.5)	48 (31.2)	< 0.001	25.036	308 (14.1)	121 (20.2)	57 (28.9)	< 0.001	37.052
High	2490 (83.7)	2384 (84.5)	106 (68.8)			1871 (85.9)	479 (79.8)	140 (71.1)		
Organized social activities (%)				0.815	0.055				0.167	3.578
Yes	1060 (35.6)	1007 (35.7)	53 (34.4)			798 (36.6)	197 (32.8)	65 (33.0)		
No	1916 (64.4)	1815 (64.3)	101 (65.6)			1381 (63.4)	403 (67.2)	132 (67.0)		
Playing cards/mahjong (%)				0.073	3.215				< 0.001	50.688
Yes	731 (24.6)	703 (24.9)	28 (18.2)			609 (27.9)	95 (15.8)	27 (13.7)		
No	2245 (75.4)	2119 (75.1)	126 (81.8)			1570 (72.1)	505 (84.2)	170 (86.3)		
Neighborhood interaction (%)				0.001	11.306				0.001	14.778
Yes	2708 (91.0)	2580 (91.4)	128 (83.1)			2006 (92.1)	535 (89.2)	167 (84.8)		
No	268 (9.0)	242 (8.6)	26 (16.9)			173 (7.9)	65 (10.8)	30 (15.2)		
Physical activities (%)				0.002	10.038				0.001	13.263
Yes	2407 (80.9)	2298 (81.4)	109 (70.8)			1792 (82.2)	472 (78.7)	143 (72.6)		
No	569 (19.1)	524 (18.6)	45 (29.2)			387 (17.8)	128 (21.3)	54 (27.4)		
Sex (%)				0.009	6.740				< 0.001	80.783
Male	1256 (42.2)	1207 (42.8)	49 (31.8)			1026 (47.1)	180 (30.0)	50 (25.4)		
Female	1720 (57.8)	1615 (57.2)	105 (68.2)			1153 (52.9)	420 (70.0)	147 (74.6)		
Age (%)				< 0.001	187.790				< 0.001	884.045
60–69	1929 (64.8)	1885 (66.8)	44 (28.6)			1715 (78.7)	170 (28.3)	44 (22.3)		
70–79	868 (29.2)	803 (28.5)	65 (42.2)			444 (20.4)	335 (55.9)	89 (45.2)		
≥ 80	179 (6.0)	134 (4.7)	45 (29.2)			20 (0.9)	95 (15.8)	64 (32.5)		
Marital status (%)				< 0.001	20.297				< 0.001	186.782
Single/ divorced/ separated/ widowed/ spinsterhood	504 (16.9)	457 (16.2)	47 (30.5)			250 (11.5)	174 (29.0)	80 (40.6)		
Married	2472 (83.1)	2365 (83.8)	107 (69.5)			1929 (88.5)	426 (71.0)	117 (59.4)		
Educational attainment (%)				< 0.001	15.280				< 0.001	203.165
Illiteracy	1679 (56.5)	1569 (55.6)	110 (71.4)			1059 (48.6)	466 (77.7)	154 (78.2)		
Primary school	894 (30.0)	866 (30.7)	28 (18.2)			765 (35.1)	98 (16.3)	31 (15.7)		
≥ junior school	403 (13.5)	387 (13.7)	16 (10.4)			355 (16.3)	36 (6.0)	12 (6.1)		
Work status (%)				0.001	14.365				< 0.001	120.396
No work	1036 (34.8)	975 (34.5)	61 (39.6)			1022 (46.9)	260 (43.3)	100 (50.8)		
Retired	1382 (46.4)	1300 (46.1)	82 (53.3)			496 (22.8)	51 (8.5)	11 (5.6)		
Still working	558 (18.8)	547 (19.4)	11 (7.1)			661 (30.3)	289 (48.2)	86 (43.6)		
Living arrangements (%)				< 0.001	32.642				< 0.001	73.708
Living with others	2761 (92.8)	2636 (93.4)	125 (81.2)			2072 (95.1)	528 (88.0)	161 (81.7)		
Living alone	215 (7.2)	186 (6.6)	29 (18.8)			107 (4.9)	72 (12.0)	36 (18.3)		
Bathroom Facilities (%)				< 0.001	18.365				< 0.001	57.508
Yes	2717 (91.3)	2591 (91.8)	126 (81.8)			2041 (93.7)	509 (84.8)	167 (84.8)		
No	259 (8.7)	231 (8.2)	28 (18.2)			138 (6.3)	91 (15.2)	30 (15.2)		
Smoking status (%)				0.035	4.456				< 0.001	39.746
Never	2339 (78.6)	2207 (78.2)	132 (85.7)			1653 (75.9)	507 (84.5)	179 (90.9)		
Current	637 (21.4)	615 (21.8)	22 (14.3)			526 (24.1)	93 (15.5)	18 (9.1)		
Alcohol use (%)				0.033	4.539				< 0.001	39.359
Never	2403 (80.7)	2268 (80.4)	135 (87.7)			1704 (78.2)	515 (85.8)	184 (93.4)		
Current	573 (19.3)	554 (19.6)	19 (12.3)			475 (21.8)	85 (14.2)	13 (6.6)		
BMI (%)				0.017	10.227				< 0.001	57.048
Underweight	140 (4.7)	125 (4.4)	15 (9.7)			74 (3.4)	41 (6.8)	25 (12.7)		
Normal	1727 (58.0)	1641 (58.2)	86 (55.9)			1241 (57.0)	371 (61.9)	115 (58.4)		
Overweight	953 (32.0)	910 (32.2)	43 (27.9)			744 (34.1)	164 (27.3)	45 (22.8)		
Obesity	156 (5.3)	146 (5.2)	10 (6.5)			120 (5.5)	24 (4.0)	12 (6.1)		
Cognitive status (%)				0.002	10.015				< 0.001	112.250
Impairment	142 (4.8)	126 (4.5)	16 (10.4)			57 (2.6)	50 (8.3)	35 (17.8)		
Normal	2834 (95.2)	2696 (95.5)	138 (89.6)			2122 (97.4)	550 (91.7)	162 (82.2)		
Number of chronic diseases (%)				< 0.001	17.020				< 0.001	35.708
0	933 (31.4)	904 (32.0)	29 (18.8)			735 (33.7)	153 (25.5)	45 (22.8)		
1	1085 (36.4)	1030 (36.5)	55 (35.7)			802 (36.8)	217 (36.2)	66 (33.5)		
≥ 2	958 (32.2)	888 (31.5)	70 (45.5)			642 (29.5)	230 (38.3)	86 (43.7)		

^a^ Continuous variables are shown as median (IQR) and categorical variables are shown as frequency (%)

^*^ Across the groups of BADL/IADL disability trajectory, for continuous variables, the p-trend was computed from the Mann–Whitney U test and the Kruskal–Wallis H test. For categorical variables, the p-trend was computed from the Chi-Square test

**Table 3:** Association between trajectories of BADL/IADL disability trajectories and social participation ^a^

	Model 1^b^ OR (95% CI)			Model 2^c^ OR (95% CI)			Model 3^d^ OR (95% CI)		
	BADL	IADL		BADL	IADL		BADL	IADL	
	Increase	Moderate	Increase	Increase	Moderate	Increase	Increase	Moderate	Increase
Extensive social participation	0.73 (0.61–0.87) ***	0.76 (0.69–0.84) ***	0.66 (0.56–0.77) ***	0.83 (0.69–1.00) *	0.86 (0.76–0.96) **	0.77 (0.64–0.92) **	0.83 (0.68–1.00) *	0.84 (0.75–0.95) **	0.77 (0.64–0.93) **
Extensive social participation level									
Low	Ref	Ref	Ref	Ref	Ref	Ref	Ref	Ref	Ref
High	0.41 (0.28–0.58) ***	0.65 (0.52–0.82) ***	0.40 (0.29–0.56) ***	0.46 (0.31–0.67) ***	0.68 (0.51–0.89) **	0.44 (0.30–0.65) ***	0.45 (0.31–0.66) ***	0.67 (0.51–0.88) **	0.45 (0.31–0.67) ***
Different types of social participation									
Organized social activities	1.18 (0.82–1.69)	0.98 (0.80–1.19)	1.10 (0.80–1.52)	1.25 (0.85–1.82)	1.00 (0.79–1.26)	1.14 (0.79–1.64)	1.26 (0.86–1.85)	0.99 (0.79–1.25)	1.11 (0.77–1.61)
Playing cards/mahjong	0.66 (0.42–0.99)	0.49 (0.38–0.62) ***	0.40 (0.26–0.61) ***	0.95 (0.59–1.50)	0.78 (0.58–1.04)	0.71 (0.44–1.15)	0.95 (0.58–1.51)	0.74 (0.55–0.98) *	0.75 (0.46–1.23)
Neighborhood interaction	0.50 (0.32–0.81) **	0.77 (0.56–1.04)	0.54 (0.35–0.83) **	0.60 (0.38–1.01) *	0.85 (0.59–1.22)	0.63 (0.38–1.03)	0.59 (0.37–0.99) *	0.86 (0.60–1.23)	0.65 (0.39–1.07)
Physical activities	0.57 (0.40–0.84) **	0.80 (0.63–1.01)	0.58 (0.41–0.82) **	0.58 (0.39–0.88) **	0.73 (0.56–0.96) *	0.57 (0.38–0.84) **	0.58 (0.39–0.87) **	0.72 (0.55–0.95) *	0.57 (0.38–0.85) **

^a^ OR = Odds ratio; CI = Confidence interval. dependent variable: BADL/IADL disability trajectory (reference: stable group)

^b^ The associations in Model 1 are unadjusted

^c^ The associations in Model 2 are adjusted for age, sex, marital status, educational attainment, work status, living arrangements and bathroom facilities

^d^ The associations in Model 3 are additionally adjusted for smoking status, consuming alcohol status, BMI, cognitive status, and the number of chronic diseases

^*^P < 0.05, **P < 0.01, ***P < 0.001

### Association between social participation and the risk of BADL/IADL disability

Table 2 presents the characteristics distinguishing disability trajectories in older people. Comparatively, participants in the stable group of BADL trajectory and the stable group of IADL trajectory exhibited higher scores in extensive social participation (all *P* < 0.05), with values of 2.00 (2.00, 3.00) and 2.00 (2.00, 3.00), respectively.

Regarding the BADL trajectory, older people who did not engage in neighborhood interactions or physical activities had a higher risk of BADL disability. Furthermore, significant differences were observed in all variables about sociodemographic characteristics and health-related factors within the BADL disability groups (all *P* < 0.05). As for the IADL trajectory, apart from organized social activities, all variables were associated with IADL disability (all *P* < 0.05).

Table 3 presents the results of logistic regression for BADL and IADL disability trajectories, with the respective stable trajectory groups as the reference. Model 1, without adjusting for any covariates, the findings revealed that for every point increment in extensive social participation score, the odds of older individuals being assigned to the increase trajectory group in BADL decreased by 27% (OR = 0.73, 95% CI = 0.61–0.87). In IADL, this increase reduced the odds of being assigned to the moderate trajectory group by 24% (OR = 0.76, 95% CI = 0.69–0.84) and to the increase trajectory group by 34% (OR = 0.66, 95% CI = 0.56–0.77). Model 2, which adjusted for sociodemographic characteristics, demonstrated a slight attenuation in these associations, but they remained significant. Model 3, which adjusted for all covariates, revealed results broadly consistent with Model 2. Specifically, in BADL, every point increment in extensive social participation score decreased the odds of older people being assigned to the increase trajectory group by 17% (OR = 0.83, 95% CI = 0.68–1.00). In IADL, it reduced the odds of being assigned to the moderate trajectory group by 16% (OR = 0.84, 95% CI = 0.75–0.95) and to the increase trajectory group by 23% (OR = 0.77, 95% CI = 0.64–0.93). For both BADL and IADL, in all models mentioned, older adults with high social participation levels have a probability reduction of at least 40% in being assigned to the increase trajectory group.

In addition, the association analysis between the four types of social participation and BADL and IADL disability trajectories revealed similar negative correlations. Specifically, after adjusting for all variables, the results showed that older people who engaged with neighbors and participated in physical activities were 41% (OR = 0.59, 95% CI = 0.37–0.99) and 42% (OR = 0.58, 95% CI = 0.39–0.87) less likely to be in the increase trajectory group in BADL. In the context of the IADL trajectory, participation in physical activities decreased the odds of older people being classified into the moderate trajectory group and the increase trajectory group by 28% (OR = 0.72, 95% CI = 0.55–0.95) and 43% (OR = 0.57, 95% CI = 0.38–0.85), respectively. Conversely, playing cards or mahjong decreased the odds of older people being classified into the moderate trajectory group by 26% (OR = 0.74, 95% CI = 0.55–0.98).

## Discussion

In this study, we utilized six waves of longitudinal data from a 7-year prospective community-based cohort spanning 2015 to 2022. Employing GBTM, we fitted BADL and IADL disability trajectories separately for older people aged 60 and above. Two distinct BADL disability trajectories were identified as stable (94.8%) and increase (5.2%), while IADL disability trajectories exhibited three distinct categories: stable (73.2%), moderate (20.2%), and increase (6.6%). We found a significant association between social participation and the deterioration of ADL disability trajectories. As the extensive social activity scores of older people decreased, there was an increasing tendency for BADL disability trajectories to be attributed to the increase trajectory group. Additionally, a lower level of extensive social participation scores was linked to a higher probability of IADL disability trajectories belonging to either the moderate or increase trajectory groups.

The findings of this study are in line with earlier studies that have classified IADL disability trajectories into three categories [16, 31]. However, a previous analysis of CHARLS data from 2011 to 2018 using group-based modeling identified three typical BADL disability trajectories [35]. Lee et al*.* identified four distinct trajectories of BADL disability among older adults aged ≥ 75 years over 6 years period in Korea: low, moderate, high, and progressive [12]. The reasons for the differences in trajectory categories across studies may be multifaceted, such as the age composition of participants, differences in ADL measurement scales, and local cultural and lifestyle differences [36].

Our study revealed that both BADL and IADL were primarily associated with the stable groups, which were the largest. Within the moderate and increase groups, initial disabilities were prevalent. Overall, older people in these groups demonstrated a less favorable baseline for IADL self-care abilities compared to BADL. Moreover, their disability levels appeared to be more pronounced in the later stages. This phenomenon may be attributed to the potential influence of extended lifespan on the expansion of IADL disability, especially among older people who are frailer and continue to endure disability for an extended period [37]. In addition, the increase group in the BADL disability trajectory model was the smallest among trajectory groups. A possible explanation for this finding is that most older adults maintained their independence in ADL at the sixth follow-up, and the elderly individuals included in this study were relatively younger. Other studies have indicated that BADL disability usually occurs later than IADL disability [8, 38], with difficulties typically emerging after the age of 75 [7]. A Danish longitudinal study in functional ability changes with an 8-year follow-up among 70- to 95-year-olds found that 13% and 9% of the older adults showed spontaneous improvement in functional ability in the first and second rounds of surveys, respectively [38]. Our research did not identify a complete high recovery group, possibly due to differences in sample size, participant age, and follow-up duration. However, the increase trajectory group in IADL exhibited improvements in functional ability by the sixth follow-up wave, with a significantly higher proportion of the oldest age group (≥ 80 years) among IADL disability trajectory groups. This might be because ADL disability among older adults is not inherently a fixed or deteriorating condition, and limitations in IADL may be partially reversible by good quality of life and vigorous physical activity [11, 12]. Consequently, it is imperative to implement early intervention measures during systematic training, rehabilitation, and health promotion efforts among old adults.

To the best of our knowledge, this study is one of the few to employ the GBTM model to simultaneously explore the relationships between social participation and the trajectories of BADL and IADL disability in Chinese older adults, where informal social participation is more prevalent than formal engagement, with a higher proportion [39]. Previous studies have shown that extensive social participation was indeed negatively associated with both BADL and IADL disability in older people [16, 40], which is in line with the results of our study. Social participation fosters psychological resilience in older people, encourages healthier behaviors, and enhances cognitive and physical functioning [26]. These positive factors, in turn, may contribute to sustained engagement in social activities over the long term [41].

Prior studies have suggested that frequent participation in various social activities was significantly associated with prolonged overall survival in older people [19, 23]. Our further analysis revealed variations in the significant associations between different categories of social participation and the trajectories of BADL and IADL disabilities. To be more specific, regular physical exercise effectively improves the trajectories of BADL and IADL disability, reducing the likelihood of older individuals being categorized into the increase group by at least 40%, in line with Zhang’s conclusion [42], highlighting the importance of being physically active in healthy aging [30]. Furthermore, socializing with neighbors contributes to the preservation of BADL functioning, whereas playing cards or mahjong can delay the onset of IADL disability. However, according to the CLHLS, the odds ratio for both informal social activities (including playing cards or mahjong) and exercise among China’s oldest old have decreased since 1998, who have become more sedentary and solitary in the past two decades [43]. Therefore, health policymakers should redirect their focus toward the development of effective lifestyle interventions and the provision of a broader spectrum of activities that promote engagement among older individuals, with particular emphasis on encouraging their involvement in physical and informal social activities.

However, we did not observe any association between organized social participation and ADL, which contrasts with the findings of a cohort study conducted in Singapore [44]. This divergence could be ascribed to organized social activities, potentially influenced by physical exercise and cognitive ability, where distinct social activities may follow divergent mediating pathways [45]. In China, civil society is still in its early stages of development, resulting in a relatively limited array of formal social activities organized by the community [23], which underscores the necessity for future research endeavors aimed at enhancing community services.

### Strengths and limitations

A significant strength of our study lies in investigating the association between older adults’ social participation and the trajectories of BADL and IADL disability using longitudinal data. This insight can guide early intervention strategies for high-risk disability older adults to promote active social participation, which is also conducive to maintaining their function and slowing down the rate of decline in functioning. There are also several limitations to our study. First, this study assessed older Chinese adults living in a major city, Shanghai, thus potentially limiting the generalizability of our results. Second, there may be a bidirectional relationship between social participation and ADL functioning, where a lack of active social engagement could be an early manifestation of ADL disability [40, 41]. Although we used longitudinal data to consider the interplay between social participation and ADL abilities, we cannot completely eliminate the possibility of reverse causality. Moreover, our analysis did not evaluate the duration of an individual’s social participation [46]. Future research should measure social participation and health at multiple time points, establish trajectories of social participation, and conduct a dual-trajectory analysis of social activities and ADL abilities. Third, although we estimated the impact of different types of social participation on ADL disability trajectories, more specific aspects of social participation, such as the content and duration of relevant activities within organized social participation, including religious activities, were not investigated in detail at baseline. Finally, out of the 4050 individuals at baseline, 866 either passed away or were lost to follow-up in 2022. Consequently, we were unable to assess the ADL abilities of these individuals, potentially leading to an underestimation of ADL disability among those who had passed away.

## Conclusion

Chinese community-dwelling older adults exhibited two distinct BADL disability trajectories and three IADL disability trajectories. Active participation in social activities significantly improved the disability trajectories of Chinese older adults. For older adults at high risk of disability, policymakers should promptly implement appropriate social interventions.

### Supplementary Information

Below is the link to the electronic supplementary material.

Supplementary file 1 (PDF 203 KB)

## Data Availability

Datasets used during the current study are available from the corresponding author on reasonable request.
