# Supplementary material for: Effect of social participation on the trajectories of activities of daily living disability among community-dwelling older adults: a 7-year community-based cohort
Source: Aging Clin Exp Res. 2024 May 7;36(1):104. doi: 10.1007/s40520-024-02758-y (PMC11076373; doi:10.1007/s40520-024-02758-y)
Supplement: Supplementary file 1 — (PDF 203 KB) [file 40520_2024_2758_MOESM1_ESM.pdf]

Supplementary Material

Supplementary Table 1 ADL disability at each wave by baseline social participation [a].

Variable	ADL disability [*]	Extensive social participation	Extensive social participation level		Different types of social participation			
			Low	High	Organized social activities	Playing cards mahjong	Neighborhood interaction	Physical activities
2015 wave	182 (6.1)	2.00 (1.25, 3.00)	46 (25.3)	136 (74.7)	65 (35.7)	15 (8.2)	165 (90.7)	136 (74.7)
2017 wave	270 (9.1)	2.00 (1.00, 3.00)	83 (30.7)	187 (69.3)	76 (28.1)	27 (10.0)	223 (82.6)	191 (70.7)
2018 wave	298 (10.0)	2.00 (1.00, 3.00)	89 (29.9)	209 (70.1)	86 (28.9)	31 (10.4)	254 (85.2)	214 (71.8)
2019 wave	322 (10.8)	2.00 (1.00, 3.00)	87 (27.0)	235 (73.0)	92 (28.6)	30 (9.3)	278 (86.3)	236 (73.3)
2021 wave	464 (15.6)	2.00 (2.00, 3.00)	101 (21.8)	363 (78.2)	152 (32.8)	70 (15.1)	413 (89.0)	361 (77.8)
2022 wave	509 (17.1)	2.00 (2.00, 3.00)	100 (19.6)	409 (80.4)	169 (33.2)	87 (17.1)	452 (88.8)	404 (79.4)

[*] At least one ADL difficulty

[a] Continuous variables are shown as median (IQR) and categorical variables are shown as frequency (%)

Supplementary Table 2 Social participation by baseline characteristics [a].

Variable	N	Extensive social participation score					Extensive social participation level	
		0	1	2	3	4	Low	High
Total	2976	73 (2.5)	413 (13.9)	1277 (42.9)	913 (30.7)	300 (10.1)	486 (16.3)	2490 (83.7)
Sex								
Male	1256	19 (1.5)	151 (12.0)	444 (35.4)	441 (35.1)	201 (16.0)	170 (13.5)	1086 (86.5)
Female	1720	54 (3.1)	262 (15.2)	833 (48.5)	472 (27.4)	99 (5.8)	316 (18.4)	1404 (81.6)
Age								
60-69	1929	32 (1.7)	253 (13.1)	805 (41.7)	627 (32.5)	212 (11.0)	285 (14.8)	1644 (85.2)
70-79	868	30 (3.5)	127 (14.6)	393 (45.3)	246 (28.3)	72 (8.3)	157 (18.1)	711 (81.9)
⩾80	179	11 (6.2)	33 (18.4)	79 (44.1)	40 (22.4)	16 (8.9)	44 (24.6)	135 (75.4)
Marital status								
Single/ divorced/ separated/ widowed/ spinsterhood	504	24 (4.8)	76 (15.1)	214 (42.4)	141 (28.0)	49 (9.7)	100 (19.8)	404 (80.2)
Married	2472	49 (2.0)	337 (13.6)	1063 (43.0)	772 (31.2)	251 (10.2)	386 (15.6)	2086 (84.4)
Educational attainment								
Illiteracy	1679	55 (3.3)	221 (13.2)	813 (48.4)	474 (28.2)	116 (6.9)	276 (16.4)	1403 (83.6)
Primary school	894	12 (1.3)	140 (15.6)	335 (37.5)	291 (32.6)	116 (13.0)	152 (17.0)	742 (83.0)
⩾junior school	403	6 (1.5)	52 (12.9)	129 (32.0)	148 (36.7)	68 (16.9)	58 (14.4)	345 (85.6)
Work status								
No work	1036	26 (2.5)	114 (11.0)	468 (45.2)	318 (30.7)	110 (10.6)	140 (13.5)	896 (86.5)
Retired	1382	36 (2.6)	233 (16.8)	553 (40.0)	410 (29.7)	150 (10.9)	269 (19.5)	1113 (80.5)
Still working	558	11 (2.0)	66 (11.8)	256 (45.9)	185 (33.2)	40 (7.2)	77 (13.8)	481 (86.2)
Living arrangements								
Living with others	2761	63 (2.3)	389 (14.1)	1169 (42.3)	862 (31.1)	278 (10.1)	452 (16.4)	2309 (83.6)
Living alone	215	10 (4.7)	24 (11.2)	108 (50.2)	51 (23.7)	22 (10.2)	34 (15.8)	181 (84.2)
Bathroom Facilities								
No	259	5 (1.9)	31 (12.0)	128 (49.4)	66 (25.5)	29 (11.2)	36 (13.9)	223 (86.1)
Yes	2717	68 (2.5)	382 (14.0)	1149 (42.3)	847 (31.2)	271 (10.0)	450 (16.6)	2267 (83.4)

Smoking status								
Never	2339	68 (2.9)	346 (14.8)	1054 (45.1)	698 (29.8)	173 (7.4)	414 (17.7)	1925 (82.3)
Current	637	5 (0.8)	67 (10.5)	223 (35.0)	215 (33.8)	127 (19.9)	72 (11.3)	565 (88.7)
Alcohol use								
Never	2403	64 (2.7)	354 (14.7)	1079 (44.9)	711 (29.6)	195 (8.1)	418 (17.4)	1985 (82.6)
Current	573	9 (1.6)	59 (10.3)	198 (34.6)	202 (35.2)	105 (18.3)	68 (11.9)	505 (88.1)
BMI								
Underweight	140	5 (3.6)	19 (13.6)	63 (45.0)	45 (32.1)	8 (5.7)	24 (17.1)	116 (82.9)
Normal	1727	42 (2.4)	245 (14.2)	739 (42.8)	531 (30.8)	170 (9.8)	287 (16.6)	1440 (83.4)
Overweight	953	20 (2.1)	125 (13.1)	409 (42.9)	288 (30.2)	111 (11.7)	145 (15.2)	808 (84.8)
Obesity	156	6 (3.8)	24 (15.4)	66 (42.3)	49 (31.4)	11 (7.1)	30 (19.2)	126 (80.8)
Cognitive status								
Impairment	142	8 (5.6)	23 (16.2)	55 (38.7)	46 (32.4)	10 (7.1)	31 (21.8)	111 (78.2)
Normal	2834	65 (2.3)	390 (13.8)	1222 (43.1)	867 (30.6)	290 (10.2)	455 (16.1)	2379 (83.9)
Number of chronic diseases								
0	933	30 (3.2)	123 (13.2)	391 (41.9)	294 (31.5)	95 (10.2)	153 (16.4)	780 (83.6)
1	1085	26 (2.4)	147 (13.5)	458 (42.2)	338 (31.2)	116 (10.7)	173 (15.9)	912 (84.1)
≥2	958	17 (1.8)	143 (14.9)	428 (44.7)	281 (29.3)	89 (9.3)	160 (16.7)	798 (83.3)

[a] Continuous variables are shown as median (IQR) and categorical variables are shown as frequency (%)

Supplementary Table 3 Four types of social participation by baseline characteristics [a].

Variable	N	Different types of social participation			
		Organized social activities	Playing cards mahjong	Neighborhood interaction	Physical activities
Total	2976	1060 (35.6)	731 (24.6)	2708 (91.0)	2407 (80.9)
Sex					
Male	1256	515 (41.0)	496 (39.5)	1139 (90.7)	1016 (80.9)
Female	1720	545 (31.7)	235 (13.7)	1569 (91.2)	1391 (80.9)
Age					
60-69	1929	696 (36.1)	545 (28.3)	1782 (92.4)	1569 (81.3)
70-79	868	300 (34.6)	160 (18.4)	774 (89.2)	705 (81.2)
≥80	179	64 (35.8)	26 (14.5)	152 (84.9)	133 (74.3)
Marital status					
Single/ divorced/ separated/ widowed/ spinsterhood	504	181 (35.9)	94 (18.7)	452 (89.7)	396 (78.6)
Married	2472	879 (35.6)	637 (25.8)	2256 (91.3)	2011 (81.4)
Educational attainment					
Illiteracy	1679	550 (32.8)	276 (16.4)	1530 (91.1)	1377 (82.0)
Primary school	894	322 (36.0)	305 (34.1)	822 (91.9)	698 (78.1)
≥junior school	403	188 (46.7)	150 (37.2)	356 (88.3)	332 (82.4)
Work status					
No work	1036	386 (37.3)	222 (21.4)	942 (90.9)	894 (86.3)
Retired	1382	497 (36.0)	368 (26.6)	1247 (90.2)	1057 (76.5)
Still working	558	177 (31.7)	141 (25.3)	519 (93.0)	456 (81.7)
Living arrangements					
Living with others	2761	994 (36.0)	690 (25.0)	2513 (91.0)	2228 (80.7)
Living alone	215	66 (30.7)	41 (19.1)	195 (90.7)	179 (83.3)
Bathroom Facilities					
No	259	87 (33.6)	51 (19.7)	238 (91.9)	225 (86.9)

Yes	2717	973 (35.8)	680 (25.0)	2470 (90.9)	2182 (80.3)
Smoking status					
Never	2339	797 (34.1)	424 (18.1)	2123 (90.8)	1896 (81.1)
Current	637	263 (41.3)	307 (48.2)	585 (91.8)	511 (80.2)
Alcohol use					
Never	2403	811 (33.7)	486 (20.2)	2177 (90.6)	1951 (81.2)
Current	573	249 (43.5)	245 (42.8)	531 (92.7)	456 (79.6)
BMI					
Underweight	140	51 (36.4)	22 (15.7)	128 (91.4)	111 (79.3)
Normal	1727	622 (36.0)	402 (23.3)	1575 (91.2)	1397 (80.9)
Overweight	953	337 (35.4)	272 (28.5)	864 (90.7)	778 (81.6)
Obesity	156	50 (32.1)	35 (22.4)	141 (90.4)	121 (77.6)
Cognitive status					
Impairment	142	56 (39.4)	18 (12.7)	123 (86.6)	114 (80.3)
Normal	2834	1004 (35.4)	713 (25.2)	2585 (91.2)	2293 (80.9)
Number of chronic diseases					
0	933	337 (36.1)	226 (24.2)	853 (91.4)	751 (80.5)
1	1085	411 (37.9)	272 (25.1)	989 (91.2)	869 (80.1)
≥2	958	312 (32.6)	233 (24.3)	866 (90.4)	787 (82.2)

[a] Continuous variables are shown as median (IQR) and categorical variables are shown as frequency (%)
